# Spatial transmission risk during the 2007-2010 Q fever epidemic in The Netherlands: Analysis of the farm-to-farm and farm-to-resident transmission

**DOI:** 10.1371/journal.pone.0227491

**Published:** 2020-02-04

**Authors:** Aline A. de Koeijer, Thomas J. Hagenaars, Jeroen P. G. van Leuken, Arno N. Swart, Gert Jan Boender

**Affiliations:** 1 Department of Bacteriology and Epidemiology, Wageningen Bioveterinary Research, Lelystad, The Netherlands; 2 Centre for Infectious Disease Control, National Institute for Public Health and the Environment, Bilthoven, The Netherlands; FDA, UNITED STATES

## Abstract

Between 2007 and 2010 a Q fever epidemic in Dutch dairy goat farms caused a large Q fever outbreak in human residents in the southern part of the Netherlands. Here we characterize the transmission of *Coxiella burnetii*, the aetiological agent of Q fever, between infected and susceptible dairy goat farms by estimating a spatial transmission kernel. In addition, we characterize the zoonotic transmission of *C*. *burnetii* by estimating the spatial kernel for transmission from infected farms to neighbouring residents. Whereas the range of between-farm transmission is comparable to the scale of the Netherlands, likely due to long-range between-farm contacts such as animal transport, the transmission risk from farms to humans is more localized, although still extending to 10 km and beyond. Within a range of about 10 km, the transmission risk from an infected goat farm to a single resident is of the same order of magnitude as the farm-to-farm transmission risk per animal in a receiving farm. We illustrate how, based on the estimated kernels, spatial patterns of transmission risks between farms and from farms to residents can be calculated and visualized by means of risk maps, offering further insight relevant to policy making in a one-health context.

## Introduction

From 2007 until 2010 the Netherlands experienced a human Q fever outbreak [1]. This was referred to as the largest outbreak of Q fever ever reported in the literature [2]. Q fever is a zoonosis caused by the intracellular Gram-negative bacterium *Coxiella burnetii*, and is prevalent throughout the world [3, 4]. Domestic ruminants are considered to be the main reservoir causing Q fever in humans [3]. There is still an incomplete understanding of the maintenance of *C*. *burnetii* within the animal reservoir and its transmission pathways to humans [5]. Transmission to humans is thought to be mainly accomplished through inhalation of airborne dust particles originating from an environment contaminated with *C*. *burnetii* [4, 6–8]. Indeed, *C*. *burnetii* DNA is detected in inhalable size dust fractions within and around infected dairy goat farms [9] as well as in indoors and outdoors air samples from an infected dairy sheep farm [10]. Furthermore, it is demonstrated that *C*. *burnetii* bacteria excreted in the environment can persist over 150 days as extracellular infectious particles in dust [11]. In an early analysis of part of the Dutch 2007–2010 outbreak it is found that people living within 2 km of an affected dairy goat farm have a strongly increased risk of Q fever compared with those living more than 5 km away [12]. In an analysis that do not distinguish between infected and non-infected goat farms, it is observed that the human risk of becoming a reported Q fever case decreases for increasing distances of the residential location to the nearest goat farm [13]. More recently, descriptive spatial analyses and cluster analyses of the full epidemic data are reported [14], yielding results consistent with the earlier studies. In other countries, Q fever transmission from ruminant farms to neighboring residents have been reported over residential distances of 400 meter [15] up to 11 miles (18.3 km) [16].

The aim of this paper is to provide what is still lacking for the Dutch 2007–2010 epidemic: a more complete quantification of the spatial transmission of *C*. *burnetii*, both between farms and from farm to resident. First, we characterize the transmission of Q fever between infected and susceptible dairy goat farms by estimating a spatial transmission kernel [17] from the outbreak data. This kernel describes the overall probability of between-farm transmission, i.e. avoiding the need to quantify the contributions of individual possible transmission routes, as a function of the distance between the two farms. It reflects the transmission risk between an average infected and susceptible goat farm and forms a cornerstone for predictive calculations of transmission risks, e.g. in the form of a transmission risk map [18], and of between-farm spread scenarios. Second, we characterize the zoonotic transmission of *C*. *burnetii* by estimating the spatial kernel for transmission from infected farms to neighboring residents. This kernel is a phenomenological description of the probability of transmission from an average infected goat farm to an average nearby resident, as a function of the residential distance to the infected farm. It is a building block for predictive calculations of zoonotic transmission risks.

## Materials and methods

**Fig 1:**
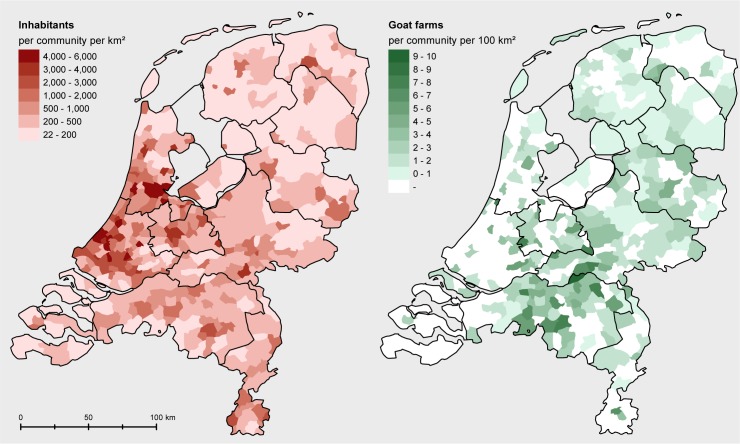
Human population density data (left panel) and the density of farms with at least 50 goats (right panel) at municipality level (the 2009 contour map of the Netherlands, source: CBS and de afdeling Geo-informatie van het Kadaster https://www.cbs.nl/nl-nl/dossier/nederland-regionaal/geografische-data/wijk-en-buurtkaart-2009).

We use spatial location data for the Dutch goat farms of November 2009, made available by the Ministry of Economic Affairs, Agriculture and Innovation (currently Ministry of Agriculture, Nature and Food Quality), and in our analysis include all goat farms with at least 50 animals. As we are interested quantifying transmission between commercial goat farms (mostly dairy goat farms), we exclude from our analysis the many farms that keep goats only on a small scale. We do this by removing from the dataset all farms with less than 50 goats. This reduced the number of farms to 404; an impression of the spatial distribution of these farms across The Netherlands is given in Fig 1. The minimum number of 50 goats is commonly used in The Netherlands as a means to select for commercial goat farms. In particular, farms with less than 50 goats were not taken into account in a bulk tank milk monitoring (BTM) program for Q fever (see below). Data on Q fever status were made available from the Animal Health Service. Surveillance and monitoring evolved during the epidemic, and an overview of legal monitoring (and control) measures is given by [5]. In summary, in the first years of the epidemic Q fever positive farms were only identified based on high abortion rates (i.e., abortion storm) notified to the Animal Health Service [19]. On 1 October 2009, a mandatory bulk tank milk monitoring was started by the Netherlands Food and Consumer Product Safety Authority, in which initially BTM samples were taken every two months, with PCR positivity of BTM becoming a notification criterion of Q fever. For farms with a notified suspicion, official Q fever infected status resulted if PCR positive results were obtained by the Dutch reference lab. In 2008, a non-systematic bulk tank monitoring program took place [20, 21]. For our analysis we identified goat farms on which the *Coxiella* bacteria was present at any time during the period 2006–2010 from four different information streams: Farms that were found positive after notifying an abortion storm, farms with positive BTM samples in the mandatory monitoring, farms with positive BTM samples in the non-systematic monitoring, and farms with positive results on vaginal swabs collected in a field study [22]. We chose to consider as Q fever source all farms with at least one positive test result in any of these four databases (based on PCR and/or serology). For lack of systematic bulk tank monitoring before October 2009 it was difficult to estimate when each infected farm became infected based on the data. In fact, a majority of infected farms was only first found positive in 2008, based on a BTM sample, and it was thus unknown how much earlier *C*. *burnetii* was introduced. For that reason we did not attempt in our analyses to assign a specific infectious period length to each of the infected farms. Instead we worked with a model considering only the average cumulative transmission (probability) across an unspecified infectious period. The analyses did however take into account an approximate chronology by assuming that infected farms could have infected only infected farms and human residents with an equal or later day at which they were first found positive in the dataset combining the four information streams. It was assumed that an infected farm could have infected any infected farm and any infected human resident with an equal or later date irrespective of how much later this date was; infected farms for which BTM results could be interpreted as indicating a termination of infectiousness before 2010 form a small minority in the dataset and thus for simplicity modelling such detail was avoided.

Human population density data at the six-digit zip code level (PC6 area, i.e. street-level) were obtained from Statistics Netherlands (CBS). A plot of this data at municipality level is given in Fig 1 next to a municipality level plot of the density of farms with at least 50 goats. Data of notified human cases, including their residential addresses at the PC6-level and dates of disease onset, were gathered by the Municipal Health Services (MHS) of Utrecht & Midden-Nederland, Brabant-zuidoost, and Zuid-Limburg, and obtained from the national infectious disease surveillance system maintained by the National Institute for Public Health and the Environment (RIVM). For the analysis of farm-to-human transmission, the centroid of the PC6 area of individual residents was used to approximate their residential location. It was assumed that an infected farm could infect patients with a date of disease onset after the starting date of the infected farm as defined above.

We modelled the probability that a given susceptible goat farm was infected by a given infected farm (cumulatively across its period of infectiousness) as a function *p*(*r*) that only depends on the straight-line distance *r* between two given farms. Likewise, we modelled the probability that a given human individual was infected by a given infected farm and subsequently diagnosed with Q fever/as Q fever positive, as a function that only depends on the straight-line distance between the farm and the residential location of the individual. In particular this implies assuming that transmission is isotropic (independent of direction) and neglecting any farm-size dependence [23]. We use the following parametrization:

p(r)=1−exp(−λ(r)),

$$\lambda (r)=\frac{\lambda_{0}}{1+{\left(\frac{r}{r_{0}}\right)}^{\alpha }}.$$

Here *λ*(*r*) is a hazard (here defined to be dimensionless) and is termed the transmission kernel. This parameterization proved suitable in earlier analyses of between-farm transmission of livestock diseases [18, 23–25]. It is flexible enough to encompass a range of possible distance dependencies of transmission and in [18, 24] it was found to produce a better fit than other parametrizations to patterns of between farm transmission. The interpretation of the three parameters in the transmission kernel is as follows: *λ*_0_ is the transmission hazard for small distances, *r*_0_ the half-value distance (*λ*(*r*_0_) = *λ*_0_/2), *α* the power. The power controls how the transmission risk scales with distance for distances (much) longer than *r*_0_. Depending on the value *α* the transmission kernel *λ*(*r*) describes local (short-range) transmission (*α*>3), intermediate-range (2<*α*<3), or global (long-range) transmission (*α*<2) [25, 26]. We did not include any farm-size dependence of farm infectiousness and farm susceptibility in our model. This was because of the computational intensity of the kernel estimation, which would further increase when allowing for additional parameters characterizing a potential farm-size dependence. We have thus restricted ourselves to fit an average transmission kernel across all infected farm sizes and across any further risk factors such as presence or absence of a notified abortion storm [14]. Previous analyses of between-transmission of other livestock diseases have shown that farm size can have a substantial impact on the infectiousness and/or susceptibility of a farm, see in particular [23] for classical swine fever. and [27] for foot-and-mouth disease. However, the same analyses indicate that whether the analysis allows for farm-dependence or not makes no significant difference to the estimated distance dependence.

The kernel parameters *λ*_0_, *r*_0_ and *α* were estimated using Maximum-Likelihood (ML), following the same approach as used in [18], and 95% confidence bounds were calculated using the likelihood-ratio test (i.e., by solving numerically for which values the univariate log-likelihood profile has dropped below its maximum by an amount corresponding to the 95% quantile of a chi-squared distribution with one degree of freedom). The calculations were done using a purpose-written script in Mathematica (Wolfram, http://www.wolfram.com). To obtain the likelihood we used that the local infection probability *p*_inf,*i*_ for an individual farm, and ${\tilde{p}}_{\inf,i}$ for a resident *i*, in the presence of a set of infectious farms *F*_inf_ can be written as:

$$\begin{array}{l} p_{\inf,i}=1-\prod_{j\in F_{\inf}}\left(1-p(r_{ij})\right)\\ \;=1-\prod_{j\in F_{\inf}}\exp(-\lambda (r_{ij})), \end{array}$$

$$\begin{array}{l} p_{\inf,i}=1-\prod_{j\in F_{\inf}}\left(1-p(r_{ij})\right)\\ \;=1-\prod_{j\in F_{\inf}}\exp(-\lambda (r_{ij})), \end{array}$$

$${\tilde{p}}_{\inf,i}=1-\prod_{j\in F_{\inf}}\exp(-\tilde{\lambda }(r_{ij})).\prod_{j\in F_{\inf}}\exp(-\tilde{\lambda }(r_{ij})),$$

and that the corresponding escape probabilities equal 1−*p*_inf,*i*_ and $1-{\tilde{p}}_{\inf,i}$. Here *r*_*ij*_ is the straight-line distance between *i* and *j*, and *λ*(*r*) and $\tilde{\lambda }(r)$ denote the farm-to-farm and farm-to-resident transmission kernel, respectively. The likelihood then reads as follows:

$$L_{\mathrm{farm}\;\mathrm{to}\;\mathrm{farm}}=\prod_{k\in F_{\mathrm{esc}}}\prod_{l\in F_{\inf}}\exp(-\lambda (r_{kl}))\prod_{i\in F_{\inf}}\left(1-\prod_{j\in F_{\inf}}\exp(-\lambda (r_{ij})H(t_{i}-t_{j}))\right)$$

$$L_{\mathrm{farm}\;\mathrm{to}\;\mathrm{resident}}=\prod_{k\in R_{\mathrm{esc}}}\prod_{l\in F_{\inf}}\exp(-\tilde{\lambda }(r_{kl}))\prod_{i\in R_{\inf}}\left(1-\prod_{j\in F_{\inf}}\exp(-\tilde{\lambda }(r_{ij})H(t_{i}-t_{j}))\right)$$

Here *F*_esc_(*F*_inf_) is the set of all farms (not) escaping from Q fever infection, *R*_esc_(*R*_inf_) is the set of all residents (not) escaping from Q fever infection, and *H*(*t*_*i*_−*t*_*j*_) denotes the indicator function which is 0 if the starting date of the farm *j* is later than the starting date of farm *i*, and 1 otherwise. This indicator function takes into account the approximate chronology of infections discussed above.

As a basis for constructing transmission risk maps [18] the local between-farm reproduction number *R*_0,*i*_, defined as the expected number of secondary cases of infection caused by infectious source farm *i*, is calculated as follows:

$$R_{0,i}=\sum_{j\neq i}p(r_{ij}).$$

We also construct resident infection risk maps by mapping the local infection probability ${\tilde{p}}_{\inf,i}$.

## Results

Some descriptive statistics of the case data are as follows. The total number of human cases in our dataset is 3777. This number and the spatio-temporal distribution of human cases is in line with data shown in Ref. [5] which shows that the vast majority of notified human cases occurred in a region in the South-East of the Netherlands. In our analysis the total number of source farms is 176. This compares to total number of 117 farms [14] that at any time had the official Q fever infected status. The difference is explained by two causes: first, only the abortion storm notifications and the notifications from the mandatory BTM sampling could impact official Q fever status (and not the non-systematic BTM program or the field study) and second, part of the notifications resulting from the mandatory BTM monitoring program were tested PCR negative by the Dutch reference lab. The median straight-line distance between source farms is 66 km, and the 95% interval is 9–198 km. The highest density of source farms occurs in the South-East of the Netherlands, in line with the spatial distribution of the official case farms provided in Ref. [5].

**Fig 2:**
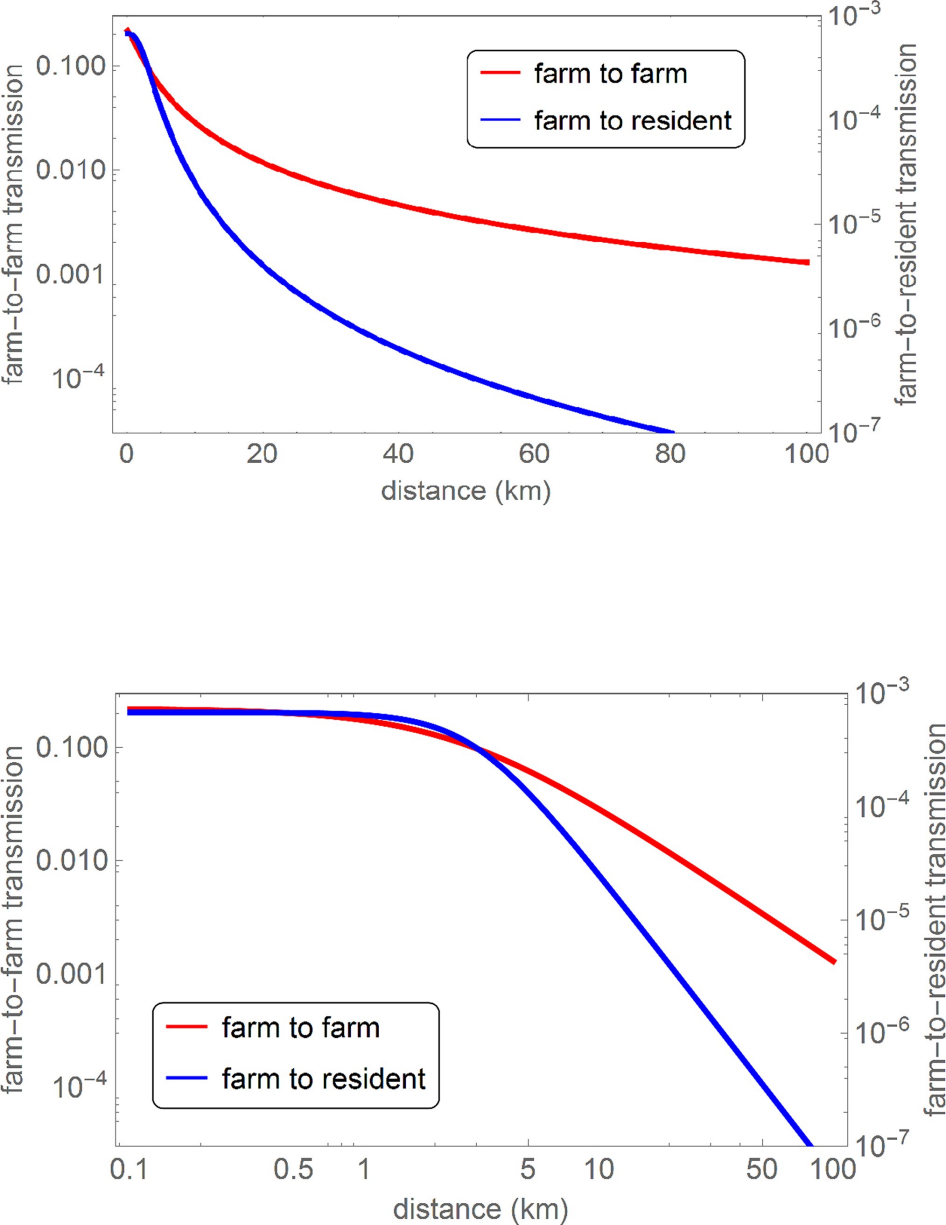
**The farm-to-farm and farm-to-resident Q fever transmission kernel as a function of the source-receptor distance, displayed on a log-log (upper panel) and log-linear (lower panel) scale.** The smooth curves are obtained by plotting the analytical expression of the transmission kernel ($\lambda (r)=\frac{\lambda_{0}}{1+{\left(\frac{r}{r_{0}}\right)}^{\alpha }}$) for the parameters in Table 1.

In Fig 2 both estimated transmission kernels are plotted, showing how the farm-to-farm and the farm-to-resident Q fever transmission risk are declining with the distance between infection source and receptor. In Table 1 the underlying parameter estimates are given. These estimates show that for farm-to-resident transmission the half-value distance *r*_0_ is around 3 km and the power *α* has a value between 2 and 3, corresponding to intermediate-range transmission. For farm-to-farm transmission the power is significantly below 2, corresponding to long-range transmission. The difference in *α* between the two kernels (a significant difference as can be seen from Table 1) corresponds to a different scaling behavior of the risk beyond distances of approximately 10 km, as is apparent most clearly from the differing constant slopes above 10 km in the upper panel of Fig 2.

**Table 1:** Estimated parameter values for the farm-to-farm and farm-to-resident Q fever transmission kernel.

	λ_0_	r_0_ (km)	α
Farm to farm	0.22 (0.074–0.76)	2.57 (0.57–7.81)	1.40 (1.11–1.76)
Farm to resident	0.00068 (0.00058–0.00081)	2.94 (2.62–3.27)	2.67 (2.59–2.76)

**Fig 3:**
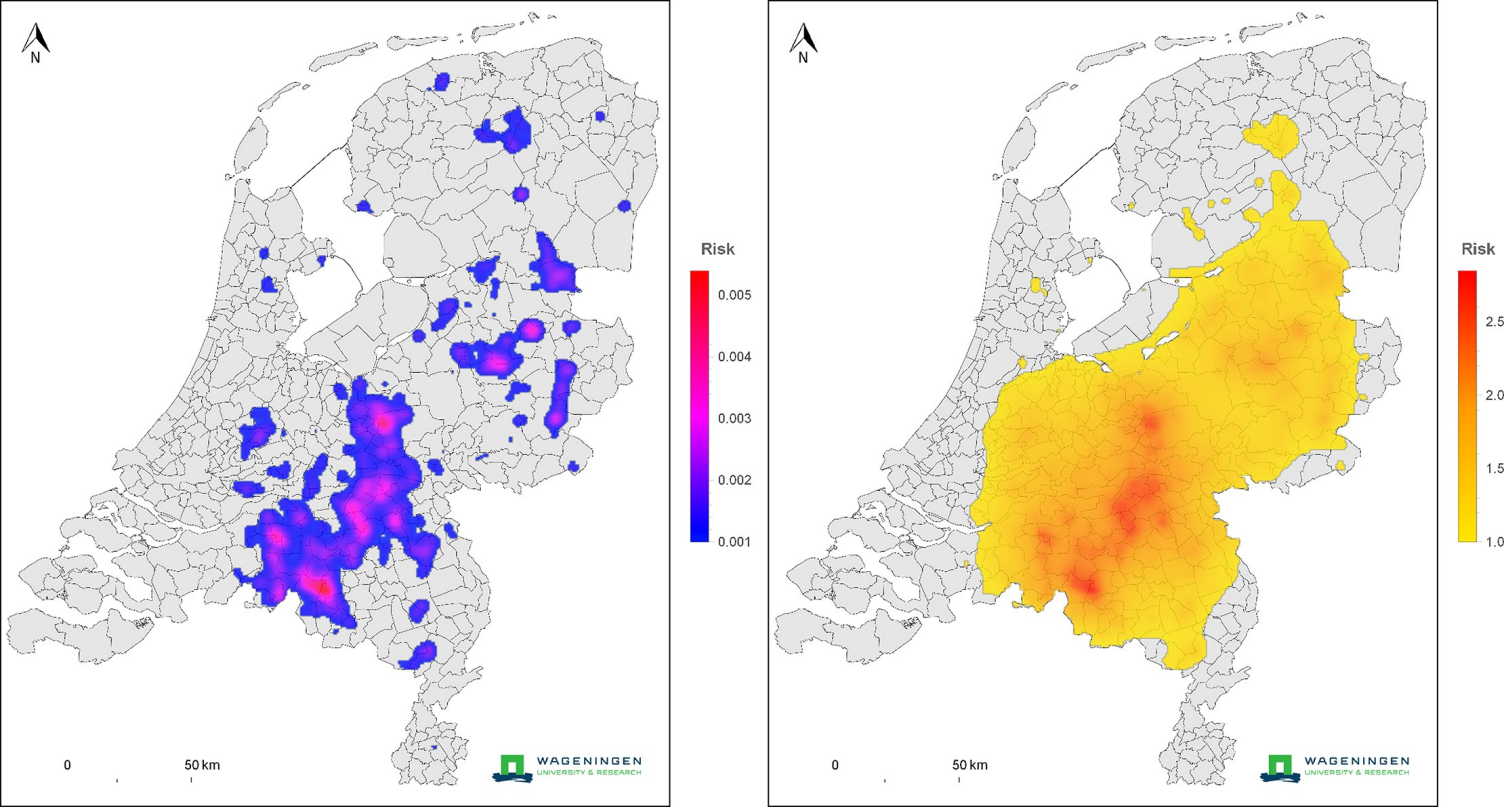
Upper panel: Farm-to-farm transmission risk map, where risk is expressed in terms of a reproduction ratio. In areas with a risk higher than 1, epidemic spread between farms may occur. Lower panel: infection risk to human residents. At each location the color indicates the potential risk to a Dutch resident at that location, for the worst case scenario that all farms are infected (the 2009 contour map of the Netherlands, source: CBS and de afdeling Geo-informatie van het Kadaster https://www.cbs.nl/nl-nl/dossier/nederland-regionaal/geografische-data/wijk-en-buurtkaart-2009).

In order to show how the quantification of transmission in terms of a transmission kernel enables the construction of predictive model extrapolations, we present two risk maps that are calculated based on the estimated kernel(s) together with farm location data for 2010. In the upper panel of Fig 3, at each location the color represents the local between-farm transmission potential, i.e. of *R*_0,*i*_ as defined in the Methods, of an average unvaccinated goat farm should it be placed at that location. In the color scale only locations with *R*_0,*i*_>1 are included, corresponding to locations where, if *C*. *burnetii* is introduced in the farm, there would be a risk of prolonged onward transmission to other goats farms. This risk map indicates that this risk of prolonged onward transmission is present in most of the Netherlands. We note that this, together with the observation that the transmission kernel incorporates long-range transmission, is in accordance with the fact that whereas the heart of the epidemic in goats was in the south, case farms occurred scattered through the whole country as is clear from the map of case farms given by Roest et al. [5]. In the lower panel of Fig 3, at each location the color represents the probability of becoming a Q fever case for a hypothetical resident at that location under the (worst-case) scenario that all goat farms in the Netherlands are affected by a Q fever outbreak. Only locations with a probability >0.001 are included in the color scale.

## Discussion

In our analyses a one-health perspective is taken by considering both the farm-to-farm and the farm-to-resident transmission. To our knowledge this is the first time that the transmission of farms to humans is quantified for a zoonotic disease by means of a spatial (transmission) kernel. This quantification provides a characterization of the distance-dependent Q fever risk from goat farm to resident that is more detailed and complete than those obtained in previous analyses based on residential distances to the nearest goat farm in 2009 and based on a spatially and temporally limited part of the epidemic in 2008 [12], and supplements descriptive spatial analyses of the full epidemic data based on distance bands [14]. As is illustrated by the examples in Fig 3, our analysis also enables the construction of spatial risk maps. Such risk maps can be used to evaluate current or future risks, thus informing policy-making, most notably for contingency planning and the granting of new farming licenses. We note for completeness that the risk maps of Fig 3 do not apply to the current situation in the Netherlands, as currently all dairy goat farms are vaccinated against *C*. *burnetii* [28].

The farm-to-farm transmission kernel shows that transmission between farms has long-range components, as evidenced by the value of the kernel power (*α*<2). Such a long range is consistent with potential long-range transmission pathways such as animal movement between farms, given that the vast majority of the Q fever farms were first found positive before or shortly after movement restrictions were imposed on 1 October 2009 [5]. Values below 2 for *α* are also found for the transmission kernels of Swine Vesicular Disease before movement restrictions in Italy in 2006 and the kernel of Blue Tongue in Belgium in 2006 in (virtual) absence of movement restrictions; this is in contrast with the transmission kernels for the transmission of Foot and Mouth Disease in 2001, Classical Swine Fever in 1997–1998 and Avian Influenza in 2003 in The Netherlands [18, 23–26], for which the value of the kernel power is above 2, consistent with movement restrictions being imposed during the period analyzed.

The parameter estimates for the farm-to-resident transmission kernel show that the distance-dependent transmission risk from a Q fever farm to a nearby resident is of intermediate range (2<*α*<3). For this intermediate-range category, transmission is confined regionally but only if the density of (human) receptors declines sufficiently at longer distances [25]. Population density patterns in The Netherlands do satisfy this condition such that in this case transmission has a regional character, extending across residential distances up to 10 km and partly beyond (as can be seen from the analysis based on residential distance to the nearest Q fever farm by Commandeur et al. 2014 [14]. On the one hand, one may speculate whether the intermediate-range transmission character is caused in part by the comparatively strong survival of *C*. *burnetii* in the environment and its apparent high probability of infection even for relatively low doses [29]. On the other hand, one should keep in mind that infection of the resident may not necessarily take place at the residential location, i.e. movement by the resident around their residency and towards one of the infection sources may play a part [30], in particular for longer residential distances.

The magnitude of *λ*_0_, the hazard at short distances, of the farm-to-farm transmission risk exceeds that of the farm-to-resident transmission risk by a factor of approximately 300. We note that a large difference in magnitude is not surprising since the two kernels quantify for a different receptor scale, one being a goat farm with on average ‘more than 600’ susceptible goats [5], and the other an individual human resident. Translating the probability of farm-to-farm transmission at short distances into a farm-to-individual-goat probability based on assuming a farm of 650 goats, yields a value of *λ*_0_ = 3.4ˑ10^4^, which is of the same order of magnitude as the farm-to-resident transmission probability at short distances of 6.8 ˑ10^4^. Due to this result and the finding that the half-value range *r*_0_ of both kernels is similar (no significant difference between the two estimates in Table 1), the transmission risk from an infected goat farm to a single resident is of the same order of magnitude as that to a single goat in a neighboring dairy goat farm, for distances up to about 10 km. Whether this observation points towards a shared dominant transmission route can be assessed only using mechanistic modelling of possible transmission routes between goat farms and from goat farms to human residents and is outside the scope of this study.

We illustrate how the estimated kernels enable the construction of spatial risk maps, offering insight relevant to policy making and spatial planning with Q fever risk in mind. In order to obtain a one-health perspective on the Q fever risk we combine maps showing the transmission risk between goat farms with maps showing the ensuing risk to human residents. In the risk map in Fig 3 for the between-farm Q fever transmission risk (upper panel), the wide extent of the colored area reflects the long-range character of farm-to-farm transmission risk as formally established by the estimated power α<2. In comparison, the pattern of the infection risk to residents, mapped in the lower panel of Fig 3, is determined more strongly (although not completely) by the local density of (infected) farms. Also this observation can be explained by the estimated value of the kernel parameter α, which here corresponds to intermediate-range transmission (2<α<3). Finally, we note that our transmission kernel represents an average across local environmental conditions that could influence transmission. For an analysis of how local environmental conditions may influence the transmission risk from goats to human residents, see [21].
